# Saunders’s Medical Hand-Atlases

**Published:** 1902-12

**Authors:** 


SAUNDERS'S MEDICAL HAND-ATLASES. Atlas and Epitomé of Traumatic Fractures and Dislocations. By Professor Dr. H. HELFERICH, Professor of Surgery at the Royal University, Griefswald, Prussia. Edited, with additions, by Joseph C. Bloodgood, M. D., Associate in Surgery, John Hopkins University, Baltimore. From the fifth revised and enlarged German edition. With 216 colored illustrations on 64 lithographic plates, 190 text-cuts, and 353 pages of text. Philadelphia and London: W. B. Saunders & Co. 1902. [Cloth, $3.00 net.]

This is the fifth edition, revised, and enlarged, and all through its pages are found evidences of the handiwork of Dr. Joseph C. Bloodgood, of Johns Hopkins, the editor of the edition. Saunders has followed out the general plan first laid down for guidance in the issuing of these hand-atlases and has maintained the high standard. For an atlas of the hand series the book is an admirable one in every respect, chiefly however as regards the illustrations. There are over 200 colored plates and nearly that number of figures in the text. One hardly finds this amount of descriptive illustrating in larger and more pretentious books dealing with surgery. Such a book as this, is one which has long been needed by students and one might say without hesitation, it has been needed by some general practitioners.

The chief value of the work lies in its illustrations. They are admirably executed and there is everywhere evidence of the greatest care in the selection of subjects. The present book has been enlarged by the addition of skiagraphs and Prof. Helferich has wisely refrained from introducing many of the rare conditions which must have come into his clinic. He has confined the illustrations to those conditions which are expected in every day work; not the rarities. There is one fact which stands out boldly in the book. Prof. Helferich knows how to teach—even in a book. He has placed with his Röntgen-ray pictures, skiagraphs of normal joints, and with the normal and pathologic conditions side by side, one has a clearer understanding of what has happened and what will happen under certain circumstances.

